# Toll-Like Receptors as Novel Therapeutic Targets for Ovarian Cancer

**DOI:** 10.5402/2012/642141

**Published:** 2012-03-04

**Authors:** Maria Muccioli, Leslee Sprague, Harika Nandigam, Michelle Pate, Fabian Benencia

**Affiliations:** ^1^Molecular and Cell Biology Program, Ohio University, Athens, OH, USA; ^2^Biomedical Engineering Program, Russ College of Engineering and Technology, Ohio University, Athens, OH, USA; ^3^Department of Biomedical Sciences, College of Osteopathic Medicine, Ohio University, Athens, OH, USA

## Abstract

Ovarian cancer (OC) is an aggressive disease that affects approximately 1 in 70 women and has a poor prognosis (<50%, 5-year survival rate), in part because it is often diagnosed at a late stage. There are three main types of OC: neoplasms of surface epithelial, germ cell, or stromal origin, with surface epithelial tumors comprising about 80% of all OCs. In addition to improving diagnostics, it is necessary to develop more effective treatments for epithelial-origin OC. Here, we describe the paradoxical roles of toll-like receptor (TLR) signaling in the progression of cancer and discuss how its modulation may result in decreased tumor growth and metastasis via the attenuation of proangiogenic cytokines and potentiation of proapoptotic factors. In particular, it has been found that TLR activity can behave like a “double-edged sword”, as its signaling pathways have been implicated as having both tumor-suppressive and tumor-promoting effects. With particular emphasis on OC, we discuss the need to consider the signaling details of TLRs and associated proteins in the multiple cell types present in the tumor milieu to achieve safe and effective design of TLR-based cancer therapies.

## 1. Ovarian Cancer Disease Characteristics

Ovarian cancer (OC) is characterized by malignant transformation of ovarian epithelial, stromal, or germ line cells. It affects approximately 1 in 70 women and has a poor prognosis (<50%, 5-year survival rate) [1]. Early-stage OC presents no obvious symptoms in the majority of patients, and no effective screening method is available at the current time. As a result, OC is most commonly diagnosed at stage III or stage IV. In later stages, metastasis often occurs, which can involve the peritoneal surface and adjacent organs, lymph nodes, lungs, and liver, among other sites. For these patients, the outcome is poor, with an on average 20% long-term survival rate [1].

OC can develop as a result of genetic risk factors, such as mutations in *BRCA1 *and *BRCA2 *genes. However, less than 10% of OCs arise as a result of genetic predisposition. Other nongenetic causes, such as chronic inflammation can result in OC development as well [1]. It has been shown that a higher number of pregnancies and oral contraceptive use correlates to decreased OC risk, suggesting that limited ovulation creates an environment that is less conducive to neoplasm development. The most common tools used today in diagnostics include measurement of CA-125 levels in the serum, pelvic ultrasonography, and biopstic and histochemical analysis. The downfall of the screening methodology is that not all OCs exhibit elevated CA-125 levels, and this method has proven to be rather unreliable in OC detection. By the time clear symptoms are present, and a sonogram, biopsy, and immunohistochemistry are performed, the OC is often in its later stages.

 Primary treatments of stage III and IV OC include surgery, followed by several rounds of chemotherapy with radiation rarely used [1]. Typically, a full hysterectomy and bilateral salpingooophorectomy are performed. Chemotherapy for OC usually includes platinum- and taxane-based agents, with either three or six rounds of intravenous treatment administered postoperatively. Platinum-containing drugs, such as cisplatin act by binding to and cross linking DNA and taxane-containing drugs, such as paclitaxel affect microtubular formation. Thereby, both agents thus act on the tumor by preventing cell division.

 Major problems encountered with current treatments include chemotherapy side effects and drug resistance. Side effects often include pain, nausea, vomiting, alopecia, and neuropathy, among others. These side effects typically arise as a result of healthy cells being affected by the treatment, resulting in various organismal imbalances. In addition, resistance to the drugs can develop, most often as a result of the cells rapidly eliminating the agent [2]. In order to improve OC survival rates, it is necessary to investigate new targets and therapies, which can be administered independently or as adjuvants to the traditional methods of surgery and chemotherapy.

## 2. Inflammation and Cancer

Innate immunity is the first response to an immunological challenge, and the onset of an innate immune response against pathogens and “danger signals” is very rapid. Macrophages, granulocytes, dendritic cells (DCs), and natural killer (NK) cells are key immune cells that participate in innate immune responses. After contacting pathogens, these cells are able to eliminate them through several mechanisms, such as phagocytosis or generation of reactive oxygen or nitrogen species. DCs and macrophages are usually called phagocytes due to their capability to engulf foreign material. Pathogens are detected by phagocytes through the expression of conserved pathogen-associated molecular patterns (PAMPs) present on the cell surface of the pathogen. These molecules are detected by pattern recognition receptors (PRRs) expressed on immune cells [3]. Through PRR recognition, the phagocytes of the innate immune response are able to distinguish between self and foreign non-self cells. Some of the main PRRs involved in the innate immune response are toll-like receptors (TLRs) and NOD-like receptors (NLRs) [3–5].

An example of the innate immune response is the initiation of inflammation. A microorganism displaying PAMPs which has become resident within body tissues can be recognized by macrophage PRRs [3]. When this occurs, the phagocyte will internalize the microorganism by phagocytosis, become activated, and eliminate the microorganism. However, in addition to eliminating the microorganism, the activated macrophage will also begin to secrete proteins known as cytokines and chemokines. Cytokine and chemokine release can lead to increased vascular permeability and expression of cellular adhesion molecules, which can in turn increase neutrophil and monocyte recruitment and infiltration to the site of infection, thereby leading to an overall amplification of the inflammatory response [6, 7]. These cytokines are known as proinflammatory cytokines [8]. Some examples of proinflammatory cytokines include interleukin (IL)-1, IL-6, and and tumor necrosis factor-alpha (TNF-*α*) [8, 9]. Through cytokine and chemokine release, additional immune cells can be recruited to the area of infection and cause the classical symptoms of inflammation: swelling, redness, heat, and pain.

Infection, chronic irritation and inflammation are among the main causes for the initiation of different types of cancer [10]. Indeed, inflammatory cells can contribute to the proliferation, survival, and migration of tumor cells and also play an important role in shaping the tumor microenvironment [10]. It has been demonstrated that chronic inflammation and cancer are often interrelated [11]. Smoking for instance is associated with chronic inflammation of the lungs and with lung cancer, and alcohol abuse has been linked to inflammation and cancer of the liver and the pancreas [12]. The relationship between inflammation and cancer is complex, and inflammation can have either tumor-promoting or tumor-suppressive effects, depending on the type of inflammation. Thus, the field of tumor immunology is an important part of the ongoing efforts of improving cancer treatments.

## 3. Inflammation and the Tumor Microenvironment

Tumors are more than cancer cells, being also composed of non-tumor cells (leukocytes, endothelial cells, fibroblasts, and smooth muscle cells) and the extracellular matrix. Together with the tumor cells, they constitute the tumor microenvironment. The cytokine profile of the tumor microenvironment is largely a result of factors produced by the tumor cells themselves, nearby cells, and infiltrating white blood cells and can have profound effects on tumor progression [13]. Some cytokines can influence the tumor microenvironment in such a way that will suppress tumor development, while others can contribute to its growth and metastasis. Upregulation of IL-12, for example, will activate NK cells and cytotoxic T lymphocytes, resulting in cancer cell death [12]. In addition, induction of IL-23 leads to the production of interferons (IFNs) and other tumor-suppressive factors. Such molecules are activated as part of the antitumor immunity response and promote apoptosis of tumor cells.

In the case of chronic inflammation, the cytokine profile at the tumor microenvironment is dramatically different and is characterized by an increase in immunosuppressive cytokines, such as TNF-*α* and IL-6 [12]. These cytokines can activate pathways that result in production of other inflammatory molecules, capable of recruiting leukocytes, such as macrophages and DCs to the tumor site. These infiltrating leukocytes in turn can produce factors which aid in the promotion of angiogenesis and vascularization, subsequently contributing to tumor growth and metastasis [11].

Angiogenesis is critical to cancer growth, as it allows for growing vasculature that will provide sufficient nutrients to the tumor to promote its growth [11]. Chronic inflammation can create an environment in the tumor milieu that is conducive to the formation of new blood vessels and thereby facilitates tumor progression. In addition to proangiogenic factors that arise from leukocyte infiltration, the tumor cells themselves produce soluble factors that potentiate angiogenesis and secrete proinflammatory cytokines, which can indirectly stimulate tumor growth [12]. As angiogenesis is a relevant process in cancer progression, pathways that trigger it are being investigated for potential drug targets for development of antiangiogenic therapeutics.

A major proinflammatory switch that can subsequently result in proangiogenic stimuli is nuclear factor-kappa B (NF-*κ*B). NF-*κ*B comprises a family of transcription factors that regulates the production of various cytokines, chemokines, and antiapoptotic and stress-response factors [14]. Different NF-*κ*B proteins can bind to specific sites on the DNA to influence transcription of a multitude of inflammatory response genes. The transcription factors reside in the cytoplasm, in complex with inhibitors of NF-*κ*B (I*κ*Bs) and are released, upon phosphorylation of the I*κ*Bs by inhibitor of NF-*κ*B kinase (IKK). At this point, the I*κ*Bs become degraded and the transcription factors are able to translocate into the nucleus to regulate inflammatory responses. The NF-*κ*B proteins regulate inflammation by binding to specific DNA sites and upregulating or downregulating the amounts of histone remodeling proteins at the site. Some inflammatory genes which are upregulated by NF-*κ*B include a multitude of proinflammatory cytokines and chemokines, matrix metalloproteases, adhesion factors, cyclooxygenase 2, and inducible nitric oxide synthase. Upregulation of these molecules results in the recruitment of immune cells and the increased production of proinflammatory molecules.

It has been shown that constitutive activation of NF-*κ*B is associated with cancer progression [15]. Specifically, it can lead to promotion of angiogenesis and increased metastasis by producing chemokines, such as IL-8 that promote leukocyte infiltration and inflammation and increased levels of MMPs that promote tumor invasion of nearby tissue. Also, NF-*κ*B can upregulate factors like TNF-*α*, which can result in inhibition of apoptosis and also stimulates cell proliferation by increasing transcription of molecules such as IL-2 and granulocyte-macrophage colony-stimulating factor (GM-CSF). In addition, certain members of the NF-*κ*B family can also activate proapoptotic factors [16].

NF-*κ*B has thus been considered a major target for anticancer therapies. Often treatment results were poor, as NF-*κ*B proteins participate in many critical cell cycle functions, the broad-scale disruption of which can lead to a multitude of undesirable side effects [14]. It is thus necessary to investigate potential regulators of NF-*κ*B, which might be drug-target candidates for the development of tumor-targeted, antiangiogenic, and proapoptotic treatments.

## 4. Toll-Like Receptors and Cancer

**Figure 1:**
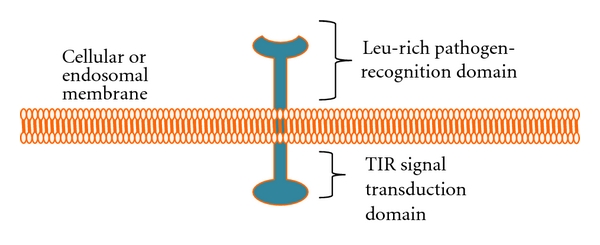
*Schematic TLR Structure. *TLRs are typically transmembrane receptors present in cellular or endosomal membranes, characterized by a leucine-rich pathogen-recognition domain and a toll-interleukin 1 receptor (TIR) signal transduction domain.

**Figure 2:**
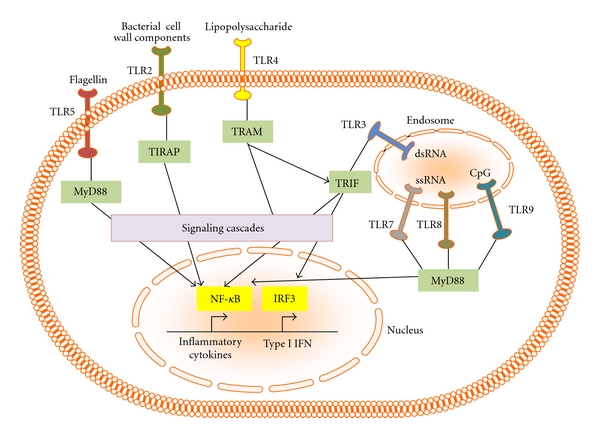
*Schematic representation of some TLR signaling adaptors some downstream effects*. Upon interaction with their ligands, TLR can induce a signaling cascade that can lead to generation of inflammatory molecules upon NF-*κ*B or IRF3 activation.

TLRs are a family of integral membrane proteins which act as sensors of invading pathogens and primarily reside in immune cells, such as DCs and macrophages [17]. Present either at the cell surface membrane or at the endosomal membrane, TLRs recognize specific PAMPs and initiate a signaling cascade to elicit an immune response. As depicted in Figure 1, all TLRs have two domains: a leucine-rich domain that senses the pathogen and a Toll-interleukin 1 receptor (TIR) domain which interacts with an adapter molecule to initiate a signaling cascade to promote an immune response. Over ten types of TLRs have been identified in vertebrates, categorization primarily based on their PAMP recognition patterns. Different TLRs recognize different PAMPs, such as protein, nucleic acid, or lipid components of bacteria or viruses. TLR4 for example, recognizes primarily bacterial components, such as lipopolysaccharide, while others, such as TLR3 recognizes dsRNA [18]. Upon recognition of the foreign pathogens, TLRs signal through adapter molecules in a signaling cascade, which ultimately results in a change in the cytokine expression patterns of those cells (Figure 2). Several adapter molecules have been characterized in the TLR family, and different TLRs signal through one of four adapters: MYD-88, MYD-88 adaptor-like (Mal), TIR-related adaptor protein inducing interferon (TRIF), or TRIF-related adapter protein molecule (TRAM) [18]. These adaptors initiate distinct signaling cascades which usually lead to activation of NF-*κ*B.

Recently, TLRs have been implicated in tumor progression. It is becoming evident that tumor cells are capable of exploiting TLR signaling pathways to their advantage [19]. It has been shown that TLR ligands can promote tumor development by stimulating inflammation, particularly through NF-*κ*B activation [18, 19]. Between tumor cells and infiltrating immune cells that express TLRs, these PRRs may be partially responsible for the constitutive activation of NF-*κ*B, which is often seen in cancers. If ligands are present to activate TLRs in the tumor milieu, this can lead to an array of cytokines capable of promoting angiogenesis. Interestingly, TLR ligands have also been shown to possess tumor-suppressive effects via activation of pathways that result in promotion of innate and adaptive antitumor responses [19]. Therefore, TLRs are significant to tumor progression and their effects on tumor development are being evaluated. They have thus become major players in the search for novel antitumor drug targets.

## 5. TLRs as Therapeutic Targets for Tumor Therapies

While TLRs are predominantly expressed in immune cells, in the last years it has been shown that they are also expressed in a variety of tumor cells where they are functional and can help shape the inflammatory profile of the tumor milieu [19]. Thus, the therapeutic use of TLR agonists has been investigated in several cancer models in order to either target tumor cells or immune cells present in the tumor microenvironment. The rationale being to either induce apoptosis of tumor cells or to activate resident immune cells that can help mount a robust antitumor response. No conclusive results have been obtained supporting the therapeutic use of TLR agonists. Positive results have been obtained by using TLR agonists as adjuvants for DC vaccination in murine models of sarcoma (TLR3/9 agonists) [20], lung cancer (TLR9) [21], and melanoma and brain cancer (TLR7/8 agonist) [22, 23]. It has also been shown that TLR agonists can enhance the efficacy of T-cell adoptive therapy by promoting a better interaction between T cells and resident activated DCs [24]. In addition, direct targeting of TLR9 in cancer cells triggered tumor cell death and an increase in survival in a xenograft model of neuroblastoma [25].

 On the contrary, TLR signaling in cancer cells can promote tumor progression. In particular, different TLR agonists were able to induce migration of human tumor colorectal, breast, lung, and glioblastoma cells, an indication of metastatic potential [26]. Further, TLR signaling has been associated with increased human myeloma and lung cells proliferation and viability [27, 28].

## 6. Ovarian Cancer Microenvironment

 These contradictory observations might be a result of the complex nature of the tumor microenvironment. As we have previously reviewed in detail, the ovarian cancer microenvironment is highly immunosuppressive [29]. For example, high levels of IL-4, IL-10, transforming growth factor beta (TGF-*β*), and vascular endothelial growth factor (VEGF) can be found in ovarian cancer ascites [30, 31]. IL-4, IL-10, and TGF-*β* can affect phagocyte function, suppressing macrophage and DC activity [32]. Indeed, DCs showing low levels of costimulatory molecules have been detected in tumors expressing high levels of VEGF [33]. These DCs are able to render T cells anergic or tolerised, thus abrogating antitumor immune responses. Interestingly, besides an immune “paralysis”, we and others have shown that tumor-associated DCs, or leukocytes expressing DC markers, are able to produce angiogenic factors and can promote neovascularization in the tumor microenvironment [34–36]. Our previous work unveiled a mechanism whereby immature DCs contribute to ovarian cancer progression by acquiring a proangiogenic phenotype in response to VEGF [36, 37]. Further, we have shown that specific depletion of tumor-associated DCs reduces ovarian cancer angiogenesis and growth [38]. Interestingly, high amounts of CD4^+^CD25^+^ regulatory T cells have been described in ovarian carcinoma and ascites, with the capability of suppressing antitumor immune responses [39, 40]. In addition, as we have previously shown, tumor endothelium can contribute to the immune suppressive status of ovarian cancer microenvironment, impairing cytotoxic T-cell infiltration [41]. On the other hand, it has been shown that ovarian cancer is capable of inducing antitumor immune responses, and that cytotoxic T-cell infiltration in ovarian cancer correlates with a better prognosis [42].

The presence of several cellular leukocyte populations in the microenvironment of ovarian cancer argues for specific targeting of tumor microenvironment components when applying TLR agonist therapies for cancer. For example, TLR agonists can be prepared for their delivery to particular cells within the tumor microenvironment [43]. This type of strategy was successfully used to activate tumor-associated DCs in ovarian cancer, promoting antitumor immune response *in vivo* [44, 45].

## 7. TLRs and Ovarian Cancer

Specifically for ovarian cancer, Zhou and colleagues [46] have shown show that TLR2, TLR3, TLR4, and TLR5 are highly expressed on the normal ovarian epithelium, as well as on neoplastic ovarian epithelial cells. Further, it has been recently shown that TLR4 is also expressed in granulosa tumor cells in the ovary [47]. In addition, it has also been demonstrated that TLR9 expression is associated with poor differentiation in ovarian cancer specimens, and that its overexpression and stimulation enhances the migratory capacity of ovarian cancer cells [48].

Importantly, TLR4 expression in ovarian cancer cells has been shown to exert protumor activities and to hamper the efficacy of paclitaxel therapy [49, 50]. This is caused by the ability of paclitaxel to interact with this TLR, activating the MyD88 signaling pathway and inducing the generation of tumor cell survival and proliferation [49–51]. This pathway points to a mechanism by which infections can promote tumor progression by stimulating cancer cells to generate inflammatory cytokines. TLR4 interacts with products of bacterial infection, but signaling through other TLRs in cancer cells can generate similar responses. To investigate this, we decided to use the MyD88 negative A2780 human ovarian cancer cell line [50, 51] to stimulate it via TLR3. This TLR is a largely endosomal PRR that recognizes dsRNA, such as viral RNA, and may also be able to recognize intrinsic dsRNA, such as that which can arise from normal events like cell lysis, for example [17, 52]. While TLR3 has no known endogenous ligands, it is suspected that various dsRNA duplexes can activate it, and synthetic dsRNA analogs which successfully bind to and stimulate TLR3 signaling have been developed [52]. One of these synthetic ligands is polyinosinic: polycytidylic acid (poly [I:C]) which has been used for many years to stimulate TLR3. As poly(I:C) has been the “gold standard” ligand for TLR3 stimulation, *in vitro* studies have been carried out using this ligand to investigate the TLR3 signaling pathways in immune cells. It has been demonstrated that poly (I:C) can directly cause apoptosis in tumor cells in a caspase-dependent manner [16]. Interestingly, it was found that certain NF-*κ*B proteins must be active for this TLR3-mediated apoptosis. Poly (I:C) has indeed been tried as an adjuvant to chemotherapy, but it was found that it was too toxic to be used for therapy [16, 52].

**Figure 3:**
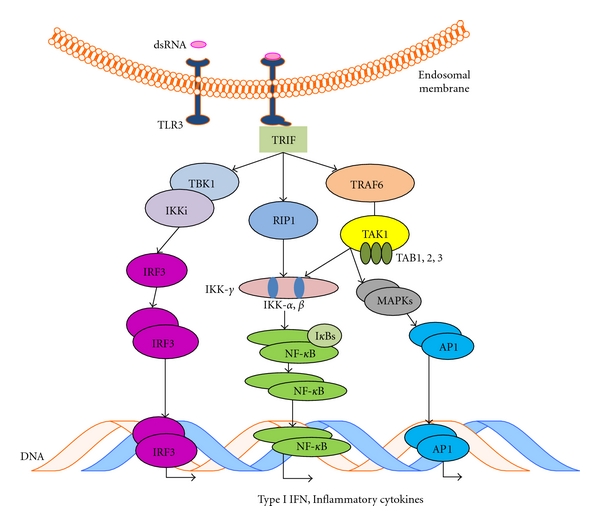
*Schematic representation of TLR3 Signaling. *TLR3 present in the endosomal particles recognizes dsRNA and interacts through cytoplasmic TRIF activating different signaling pathways.

**Figure 4:**
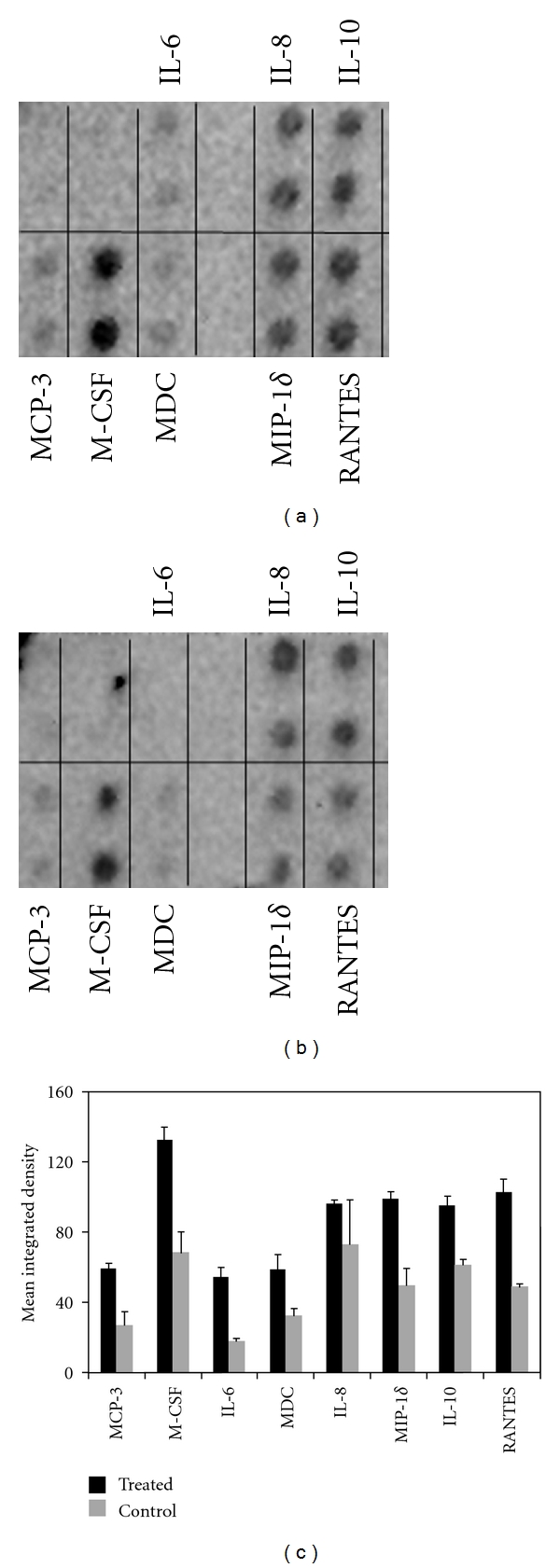
*Stimulation of A2780 ovarian cancer cells via TLR3*. A270 human ovarian cancer cells were treated for 24 h with 10 *μ*g/mL of poly(I:C) (Invivogen, San Diego, CA) admixed with lipofectamine (Invitrogen, Carlsbad, MA) following the manufacturer's instructions. Then supernatants were recovered for analysis. Supernatants from two independent experiments were pooled, and the presence of different chemokines and cytokines in poly(I:C) treated (a) or controls (b) was analyzed by using the RayBio Human Cytokine Antibody Array 3 (Raybiotech Inc, Norcross, GA) following the manufacturer's instructions. Finally, density values were analyzed by using the ImageJ program.

As shown in Figure 3, TLR3 signaling can activate NF-*κ*B in a MyD88-independent way. Upon stimulation with poly(I:C), TLR3 signals through adapter TRIF (TICAM-1) to initiate a signaling cascade that can activate transcription factors NF-*κ*B and interferon regulatory factor 3 (IRF3), as well as the JNK and p38 pathways. TRIF is the largest adaptor known of the TLR adapters and can initiate several distinct signaling cascades [52]. TRIF has an N-terminal region called the effector-driving site, which is able to recruit the TNF receptor-associated factor (TRAF) family proteins to result in the eventual activation of IRF3 and NF-*κ*B. IRF3 activation can result in the upregulation of *α*- and *β*-type interferons (IFNs) and activation of cytotoxic lymphocytes and NK cells, while NF-*κ*B can either upregulate certain cytokines and potentiate chronic inflammation and angiogenesis, or act to promote apoptosis, depending on the particular NF-*κ*B members that are activated. TRIF also has a C-terminal binding site, which can recruit factors such as receptor interacting protein (RIP-1) and Fas-associated death domain (FADD), the signaling pathways of which result in apoptosis and autophagy through the activation of the JNK and p38 pathways. Thus TLR3 signaling can contribute to tumor eradication via upregulation of IFN-*α* and IFN-*β*, CTL, and NK cell activation, and by signaling through the RIP-1/FADD pathway, whereas it can also indirectly contribute to tumor progression via activation of NF-*κ*B, which can result in proangiogenic factors and subsequent tumor progression. As shown in Figure 4, 24 h treatment of human ovarian cancer A2780 cells with 10 *μ*g/mL of poly(I:C) admixed with liposomes is able to induce upregulation of several cytokines and chemokines, IL-6 among others, which has been shown to promote tumor cell growth and survival [53]. 

Altogether these data indicate that the non-targeted use of TLR agonists for ovarian cancer therapy can generate adverse effects. Thus, targeted therapies to activate only particular components of the tumor microenvironment will be more suitable. In this context, pioneering research from Dr. Conejo-Garcia's lab has shown in a murine model of ovarian carcinoma that specifically targeting TLRs in tumor-associated antigen-presenting cells can induce a robust antitumor immune response and tumor regression [44, 45, 54, 55].

## 8. Final Remarks

 In closing, it is necessary to further elucidate all the distinct signaling pathways of TLR in both tumor and immune cells in order to develop effective and safe immunotherapies using this target. On the one hand, TLR stimulation can result in apoptosis, but on the other, it can lead in proangiogenic factors, which can stimulate tumor growth. Effectively being able to select for and specifically turn on and off these pathways is the ultimate goal. As details concerning TLR signaling in tumor cells and immune cells are clarified, it may be possible to design therapeutic agents, which target specific pathways in specific cells and create a proapoptotic, antiangiogenic tumor microenvironment, which is conducive to tumor eradication.
